# Exosomal microRNA-23a-3p contributes to the progression of cholangiocarcinoma by interaction with Dynamin3

**DOI:** 10.1080/21655979.2022.2037249

**Published:** 2022-02-24

**Authors:** Qingfeng Ni, Hai Zhang, Xiaoli Shi, Xiangcheng Li

**Affiliations:** The National Institute of Living Donor Liver Transplantation, The First Affiliated Hospital of Nanjing Medical University, Nanjing, Jiangsu, P.R. China

**Keywords:** Cholangiocarcinoma, miR-23a-3p, DNM3, exosomes

## Abstract

Cholangiocarcinoma (abbreviated as CCA) accounts for about 3% of digestive tract tumors, which is a rare disease with relatively low incidence. Herein, we firstly discovered overexpression of microRNA-23a-3p (abbreviated as miR-23a-3p) in CCA tissues, as well as cell lines via bioinformatics prediction. Next, by conducting miR-23a-3p knockdown system in HUCCT1 cells and miR-23a-3p overexpression system in RBE cells, we investigated the biological effects of miR-23a-3p. Based on our findings, inhibition of miR-23a-3p was able to prevent cancer cell proliferation via colony formation, CCK-8, as well as EdU assays. Moreover, invasion as well as migration abilities of cells was examined by transwell assay and wound healing test. Animal study further verified that knockdown miR-23a-3p slowed down tumor growth and lung metastasis. In addition, we identified cholangiocarcinoma cells transferred miR-23a-3p through exosomes by a series of assays. Functional experiments have confirmed that exosomal miR-23a-3p could benefit for cancer cell growth and metastasis, serving as a cancer promoting gene. Furthermore, we found Dynamin3 (abbreviated as DNM3) turned out to be a target of miR-23a-3p, while DNM3 was down-regulated in cholangiocarcinoma. Knockdown DNM3 accelerated cancer cell development. Collectively, our findings firstly pointed out that exosomal miR-23a-3p was conducive to the progression of cholangiocarcinoma by interaction with DNM3, which provided potential evidence for cancer treatment.

## Graphical abstract

**Figure:**
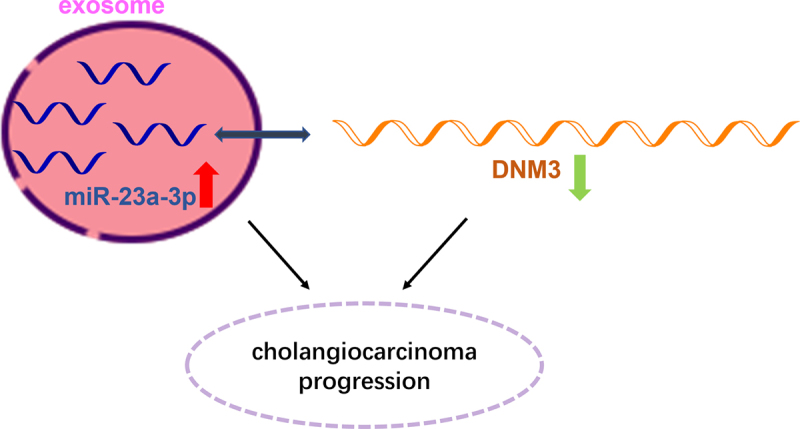

## Introduction

Epidemiological data show that cholangiocarcinoma (abbreviated as CCA) accounts for about 3% of digestive tract tumors, which is a rare disease with relatively low incidence, but the degree of malignancy is next to that of pancreatic cancer, and the mortality and recurrence rate have already reached 50–70% [1]. The efficacy of cholangiocarcinoma chemotherapy and radiotherapy was not clear, and the average survival time is less than 12 months when given gemcitabine combined with cisplatin as recommended chemotherapy treatment by the guidelines [2,3]. Therefore, cholangiocarcinoma has become a clinically difficult disease that needs to be solved, and exploring effective biomarkers and strategies are priorities for cholangiocarcinoma treatments.

Noncoding RNAs (abbreviated as ncRNAs) are a kind of sequence that can be transcribed by genome but do not participate in coding proteins, which have been proven to be novel biomarkers in various cancers. MiRNAs generally degrade mRNAs or inhibit the translation of proteins at the post-transcriptional level through complementary pairing of base sequences, and are enrolled in the processes of cell growth, differentiation, aging, apoptosis, autophagy, and metastasis [4]. There is a miRNA cluster on human chromosome 19p13.2, which is named mir-27a, mir-24-2, and mir-23a, respectively [5]. MiR-23a-3p, containing 1 exon regions, known as a cancer promoting gene, is involved in malignant cancers, such as pancreatic cancer and malignant melanoma [6–8]. Abnormal miR-23a-3p was found under many pathological conditions [9,10]. Wide researches have demonstrated that miR-23a-3p can regulate the progression of osteoarthritis and the over-expression of miR-23a-3p significantly down-regulates the expression of mRNA as well as the levels of Smad3 [11]. miR-23a-3p influences the molecular mechanism of gastric cancer cells via CCL22/PI3K/Akt axis [12]. miR-23b-3p promotes osteosarcoma by targeting VEPH1/PI3K/AKT pathway [13]. Yet the evidence of miR-23a-3p involving in cholangiocarcinoma has not been explored clearly.

Exosomes are extracellular phospholipid bilayer vesicles that are secreted by cells and with a diameter of 30–150 nm. They originate from late endosomes and are kept in multivesicular bodies, and are one of the most well-studied extracellular vesicles (abbreviated as EVs). Exosomes could be applied as carriers to encapsulate and transfer functional molecules, including protein, lipid, metabolite, DNA, mRNA, miRNA, lncRNA, etc. [14]. Existing studies have recognized that, as an important medium of intercellular communication, exosomes can transfer crucial signal molecules from source cells to target cells, mediate in tumorigenesis, progression and chemotherapy resistance [15–17], for instance, exosomal miR-128-3p could enhance chemosensitivity in colorectal cancer. Due to the wide distribution of exosome in body fluids, it is promising to be a new marker for early diagnosis of tumors.

DNM3 (Dynamin3), a family member of Dynamin, which is involved in many membrane transport functions, such as cytokinesis and phagocytosis to transport vesicle germination and cell flow [18,19]. To date, observations have indicated that DNM3 can promote tumor progression in glioma via interacting with miR-221 [20]. Decreasing cell proliferation rate and increasing apoptosis of hepatocellular carcinoma were induced by DNM3 overexpression [21]. Here, the intentions of this paper are to dig out the role exosomal miR-23a-3p plays in cholangiocarcinoma as well as its detailed mechanism.

## Materials and methods

### Bioinformatic analyses

The Cancer Genome Atlas (abbreviated as TCGA) database (http://www.cancergenome.nih.gov/dataportal) was used for the bioinformatics prediction process. The R software ‘limma’ package was used in the analysis of both cholangiocarcinoma and normal samples. The threshold values were set as p < 0.05 and |log 2 FC| > 1.

### Tissues collection

The tumor samples and normal samples of cholangiocarcinoma patients were collected at the National Institute of Living Donor Liver Transplantation, the First Affiliated Hospital of Nanjing Medical University. The hospital ethics committee approved the sample acquisition. All participants were diagnosed with cholangiocarcinoma by pathology and did not undergo chemotherapy or radiotherapy before collection. All patients signed informed consent for surgery. We freshly collected the tumor tissues as well as the matched adjacent tissues (about 2 cm from the edge of the tumor) from all the patients. We kept all the tissue specimens at −80° until experiment.

### Cell culture as well as transfection

We purchased the human cholangiocarcinoma cell line (QBC939, HUCCT1, RBE, HCCC9810) as well as human intrahepatic bile duct epithelial cell HiBEC from Shanghai Institute of Cell Biology, which was then incubated in 1640 medium that contained 10% FBS and 1% double antibody. We coloned the miR-23a-3p inhibitor as well as its mimics into pEZX-AM03 and pEZX-MR03 plasmid to regulate the expression level of miR-23a-3p. The si-DNM3 was utilized to downregulate DNM3 in HUCCT1 cell line. We seeded the cells on 24-well plates and then randomly divided them into some groups: miR-23a-3p inhibitor/inhibitor NC group, miR-23a-3p mimics/mimics NC group, as well as miR-23a-3p inhibitor + si-DNM3 group. When the cells were fused to 70%, transfection was carried out according to the instruction of lipofectamine^TM^ 3000 transfection reagent (Invitrogen).

### QRT-PCR assay

Trizol reagent (Invitrogen) was applied for RNA extraction, and then the Prime ScriptTM RT reagent Kit (TaKaRa) was applied for reverse transcription into cDNA. We stored the cDNA resulted at −20°C used for qPCR analysis. We measured the expression level of miR-23a-3p with the help of TaqMan MicroRNA Assays, and U6 was applied as the internal control. The relative expression level of the gene was calculated using 2^−Δ CT^ or 2^−ΔΔ CT^ method. The primer sequences are listed in Table 1. This experiment followed MIQE guidelines (consulting https://pubmed.ncbi.nlm.nih.gov/19246619/).

**Table 1.** The primers used in this study

Name	Sequences
miR-23a-3p forward primer	5’‑GCGATCACATTGCCAGGG‐3’
miR-23a-3p reverse primer	5’‑AGTGCAGGGTCCGAGGTATT‐3’
U6 forward primer	5’-CTCGCTTCGGCAGCACAA‐3’
U6 reverse primer	5’-AACGCTTCACGAATTTGCGT‐3’
miR-23a-3p inhibitor	5’‐GGAAAUCCCUGGCAAUGUGAU‐3’
inhibitor NC	5’-CAGUACUUUUGUGUAGUACAA‐3’
miR-23a-3p mimics	5’-AUCACAUUGCCAGGGAUUUCC‐3’
mimics NC	5’-UUGUACUACACAAAAGUACUG‐3’
si-DNM3	5’-GGGAUGUUCUAGAGAACAATT‐3’
si-NC	5’-UUCUCCGAACGUGUCACGUTT‐3’

### Western blot assay

We extracted the total protein with the help of RIPA lysate, then determined with the help of BCA kit, the matched samples were adjusted to the same concentration. After denaturation, we separated the proteins with the help of SDS-polyacrylamide gel electrophoresis and then transferred them onto PVDF membrane. We sealed the PVDF membrane for 1 h using 5% skimmed milk powder, then incubated the PVDF membrane overnight with the following primary antibodies against Bcl-2 (Abcam, ab32124, 1:1000), Bax (Abcam, ab32503, 1:1000), Vimentin (Abcam, ab137321, 1:2000), E-cadhetrin (Abcam, ab238099, 1:100), CyclinD1 (Abcam, ab134175, 1:5000), DNM3 (Abcam, ab134925, 1:1000) protein or GAPDH (Abcam, ab181602, 1:5000). We then incubated the PVDF membrane for 1 h with secondary antibody. ECL kit was used for chemiluminescence detection. The gray value of protein band was calculated using Image-Pro Plus.

### CCK-8 as well as EdU assays for cell proliferation

CCK-8 assay was conducted as follows: After culturing for 48 h, we added 10 μL of CCK-8 solution (Dojindo) to each well of 96-well plate. The cell viability of the different groups was measured on 24, 48, 72 and 96 h according to the instruction of CCK-8 kit. EdU assay was conducted as followed: We added 100 μmol/L of 1640 culture containing 50 μmol/L 5-ethynyl-2’-deoxyuridine (abbreviated as EdU) to each well then incubated them for 2 h. Then, we incubated the cells for 10 min using 2 mg/ml glycine after 30 min in 4% paraformaldehyde. After that, we washed the cells using PBS, then infused them for 10 min with 500 μL 0.5% triton-x, and then incubated them in darkness for 30 min with the Apollo staining solution. Finally, we incubated the cells for 30 min with Hoechst, then washed them twice using 0.5% triton-x, and then observed them under the fluorescence microscope.

### Colony formation assay

Cells were treated according to different groups. After 48 h, we inoculated 500 cells per well in 6-well plates, and changed the medium every 2 days. After 2 weeks, PBS was applied for washing, 4% paraformaldehyde was for fixing, then stained for 10 min with crystal violet, observed as well as photographed under microscope after drying.

### Transwell assays

Migration assay: After transfection of cells for 48 h, we put 100 μL of the cell suspension into the upper chamber with 500 μL of medium in the lower chamber. Then we incubated the cells for 12 h. We took out the chamber, gently wiped off the upper surface of the cells with a cotton swab, and then fixed them using neutral formaldehyde for hematoxylin staining. The number of migration cells was counted with the help of a microscope. Invasion assay: the method was the same as above, but invasion cell is coated with artificial base glue.

### Wound healing test

Cells were collected in the logarithmic growth period. We used Draw 200 μL tip to draw lines vertically (5 mm), then washed the cells using PBS, and then photographed and recorded immediately after fresh 1640 cell medium was changed. After 48-h culture, we photographed the cells again, then the migration distance of each group was observed and calculated.

### Exosomes separation

We purified the exosomes in the culture supernatant of cholangiocarcinoma cells using differential ultracentrifugation. Briefly, we cultured cells for 48 h in the 1640 medium that was supplemented with 10% of exosome-depleted FBS (SBI, USA); then, we collected the cell culture supernatant and spun them at 300 g for 10 min under 4°C for removal of cells and then 2000 g for 10 min under 4°C for removal of cellular debris. The obtained supernatants were followed by ultra-centrifugation at 10,000 g for 30 min under 4°C. After that, the supernatants were ultra-centrifuged at 100,000 g for 70 min under 4°C. The precipitations were resuspended in PBS, that was exosomes solution. Observed and photographed under transmission electron microscope (abbreviated as TEM), and the size of particles was analyzed and identified by using nano-particle tracking analysis method.

### TEM observation and NTA (nanoparticle tracking analyzer)

We fixed the exosomes using 1% of glutaraldehyde then stained them with 1% of phosphotungstic acid. We observed the exosomes under transmission electron microscope and photographed them. The NTA was applied via Zeta View system to observe the Brownian movement of the exosomes, and the distribution of particle size was analyzed using the Stokes-Einstein equation.

### The cellular uptake of exosomes

Exosomes were labeled proportionally according to the PKH67 dye instructions. After purification and resuspension, exosomes were mixed with the cell medium and then co-cultured with RBE cells in dark for 12 h. PBS was washed for 3 times. After fixed using 4% paraformaldehyde solution, washed using PBS for 3 times, DAPI staining for 5–10 min, and then PBS washing for 3 times. We sealed the anti-fluorescence quenching sealing tablets then took photos under a fluorescence microscope.

### Dual-Luciferase reporter assay

The 3 ‘UTR wild-type (abbreviated as WT) luciferase vector pGL3-DNM3-WT as well as mutant (abbreviated as MUT) luciferase vector pGL3-DNM3-MUT were constructed. We co-transfected PGL3-DNM3-WT with miR-23a-3p miR-NC or mimics, and at the same time co-transfected pGL3-DNM3-Mut with miR-23a-3p miR-NC or mimics into RBE cells with the help of Lipofectamine^TM^ 3000 kit. And 48 h after transfection, we measured the luciferase activity with the help of the official kit.

### Animal study

Five-week-old male BALB/C mice were stored at 22–24°C, after adaptive feeding for 3 days, we injected HUCCT1 and RBE cells (100 μL,1 × 10^7^ cells/mL PBS) transfected miR-23a-3p inhibitor and inhibitor NC or miR-23a-3p mimics and mimics NC into the right forelimb of male nude mice subcutaneously. We recorded the volume of tumor every 7 days. At day 35, dealt with the mice and tissues were weighed. Lung metastasis model: we injected transfected cells by tail vein (100 μL,2 × 10^7^ cells/mL PBS) and observed the mice under fluorescence, 6 weeks later, mice were killed and tumor tissues were split. HE staining was applied to evaluate tumor metastasis. Animal studies were in accordance with ARRIME guidelines.

### Immunohistochemistry

We cut the tumor tissue excised into tissue blocks size to fit, then stored them for 3 days in 4% of paraformaldehyde. Then, we applied PBS to wash for 1 h, and dehydrate the tissue using xylene and ethanol. We immersed the tissue blocks in wax solution overnight at a temperature of 60°C. Then, we embedded the tissue blocks with the help of embedding machine, and then cut them into slices of 5 μm in diameter. After 40°C water bath, we dried them and then stored them at the temperature of 4°C. After that, we heated the tumor tissue blocks for 15 min to 92–98°C with the help of citrate buffer, then we digested them at room temperature using hydrogen peroxide, and finally immunohistochemical stained them using relevant antibodies.

### Statistical analysis

We applied GraphPad Prism 8 as well as SPSS 19.0 software for analysis of the experimental data. The data were expressed by mean ± standard deviation ($\overset{ˉ}{x}$ ± s). We applied t-test was for the comparison between two groups, while we applied one-way analyses of variance for the comparison between two more groups. Categorical data were analyzed using the chi-square test. P < 0.05 meant the difference was statistically significant.

## Results

### Highly expressed miR-23a-3p was found in cholangiocarcinoma tissues as well as cells

**Figure 1.**
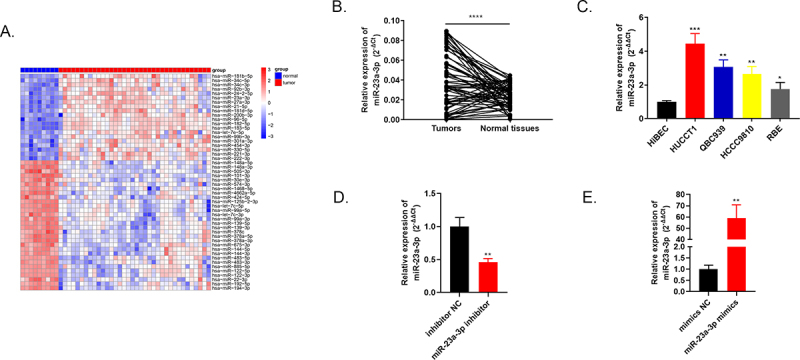
**Highly expressed miR-23a-3p was found in cholangiocarcinoma tissues as well as cells**. (a) Heatmap of primary 50 differential expressed miRNAs in 36 cholangiocarcinoma tissues in comparison to 9 adjacent tissues. (b) MiR-23a-3p was overexpressed confirmed by qRT-PCR in 60 pairs of cholangiocarcinoma tissues as well as normal tissues. (c) Higher miR-23a-3p level was observed in 4 cholangiocarcinoma cell lines (QBC939, HUCCT1, RBE, HCCC9810) compared to normal cell line HiBEC. (d) QRT-PCR detected the transfection efficacy after transfection with miR-23a-3p inhibitor or inhibitor NC in HUCCT1. (e) QRT-PCR detected the transfection efficacy after transfection with miR-23a-3p mimics as well as mimics NC in RBE.

Through bioinformatics tools, the heat map in Figure 1a exhibits top 50 differential expressed genes of 36 cholangiocarcinoma tissues compared to 9 adjacent tissues in TCGA database. One of the highly expressed genes, miR-23a-3p, was chosen as our subject. Using qRT-PCR, we discovered that miR-23a-3p was markedly up-regulated in 60 pairs of cholangiocarcinoma tissues and normal tissues (Figure 1b). Among four cancer cell lines (QBC939, HUCCT1, RBE, HCCC9810), overexpressed miR-23a-3p level was also detected compared to intrahepatic bile duct epithelial cell line of normal human HiBEC via qRT-PCR (Figure 1c). HUCCT1 cell line with the highest miR-23a-3p expression level was transfected with inhibitor of miR-23a-3p, inhibitor NC plasmids to conduct the knockdown model. The corresponding transfection efficacy was examined by qRT-PCR, the expression level of miR-23a-3p was depressed in miR-23a-3p inhibitor group in comparison to inhibitor NC group (Figure 1d). RBE cell line with the lowest miR-23a-3p level was transfected with miR-23a-3p mimics as well as mimics NC plasmids to establish the overexpression model. MiR-23a-3p expression level was elevated in miR-23a-3p mimics group in comparison to mimics NC group (Figure 1e). Moreover, we explored the relationship between miR-23a-3p expression level and the CCA patients’ clinicopathological features. The expression level of miR-23a-3p was significantly associated with lymph node metastasis (P = 0.020) as well as the tumor size (P = 0.017) in ICCA patients and was also significantly associated with lymph node metastasis (P = 0.027) in ECCA patients, as demonstrated in Table 2. Yet, it was not correlated to gender, age, tumor numbers, pathologic grade, vascular invasion, perineural invasion or TNM stage. The functional experiments were applied as follows.

**Table 2.** Correlation of miR-23a-3p expression with clinicopathological characteristics in CCA patients

Parameters	Total	ICCA		*P*-value	Parameters	Total	ECCA		Total
		High level	Low level				High level	Low level	
Age					Age				
<60	17	7	10	0.328	<60	15	8	7	0.647
≥60	21	12	9		≥60	7	3	4	
Gender					Gender				
Male	21	11	10	0.744	Male	17	9	8	0.611
Female	17	8	9		Female	5	2	3	
Tumor numbers					Tumor numbers				
1	32	15	17	0.374	1	19	9	10	0.534
≥1	6	4	2		≥1	3	2	1	
Tumor size(cm)					Tumor size(cm)				
<5	13	3	10	0.017*	<3	19	9	10	0.534
≥5	25	16	9		≥3	3	2	1	
Pathologic grade					Pathologic grade				
I+ II	12	7	5	0.485	I+ II	9	5	4	0.665
III+IV	26	12	14		III+IV	13	6	7	
Vascular invasion					Vascular invasion				
yes	27	13	14	0.721	yes	12	5	7	0.392
no	11	6	5		no	10	6	4	
Perineural invasion					Perineural invasion				
yes	25	15	10	0.087	yes	15	8	7	0.647
no	13	4	9		no	7	3	4	
Lymph node metastasis					Lymph node metastasis				
yes	15	11	4	0.020*	yes	4	4	0	0.027*
no	23	8	15		no	18	7	11	
TNM stage					TNM stage				
I+ II	18	10	8	0.516	I+ II	6	2	4	
III+IV	20	9	11		III+IV	16	9	7	0.338

### Decreasing miR-23a-3p expression could inhibit cholangiocarcinoma proliferation, invasion as well as migration

**Figure 2.**
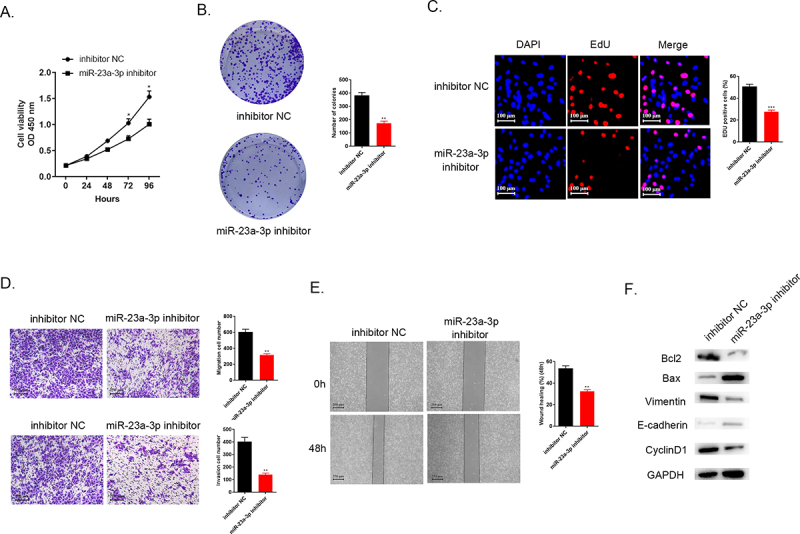
**Decreasing expression level of miR-23a-3p could inhibit cholangiocarcinoma proliferation, invasion, as well as migration**. (a) Cell viability curve of miR-23a-3p inhibitor as well as inhibitor NC groups in HUCCT1 cells measured by CCK-8. (b-c) Colony formation as well as EdU assays confirmed the cell proliferation ability of miR-23a-3p inhibitor and inhibitor NC groups in HUCCT1 cells, respectively. (d) Transwell assays evaluated cell invasion as well as migration abilities when miR-23a-3p been knockdown. (e) Wound healing test confirmed cell migration ability when miR-23a-3p been knockdown. (f) Quantitation of proteins correlated to apoptosis (Bax and Bcl-2), EMT pathway biomarkers correlated to tumor transition (Vinmentin and E-cadherin) and cell cycle protein (CyclinD1) were determined by Western blot.

After transfection, CCK-8 assays, colony formation, as well as EdU assays aimed to detect cell proliferation. The results demonstrated that the lower miR-23a-3p level in the group of miR-23a-3p inhibitor led to the inhibition of cell proliferation compared to inhibitor NC group (Figure 2a–c). Figure 2d manifests that miR-23a-3p suppression could prevent HUCCT1 cell invasion as well as migration in comparison to the control group, according to the transwell assays. Wound healing test could mimic the ability of migration in vitro. Figure 2e displays that knockdown miR-23a-3p resulted in the weaker migration ability. Furthermore, Western blot detected several proteins related to apoptosis (Bax and Bcl-2), and EMT pathway biomarkers associated with tumor transition (Vinmentin and E-cadherin) and also cell cycle protein (CyclinD1). The data in figure 2f revealed that knockdown miR-23a-3p could down-regulate apoptosis regulators Bcl-2 level and up-regulate Bax level, inhibit Vimentin expression and increase E-cadherin protein level and the decreasing CyclinD1 level was also seen in miR-23a-3p inhibitor group. The evidence collectively exhibited that down-regulation of miR-23a-3p could impede proliferation, invasion, as well as migration of cholangiocarcinoma cells in vitro.

### Increasing miR-23a-3p expression could accelerate proliferation, invasion as well as migration of cancer cells

**Figure 3.**
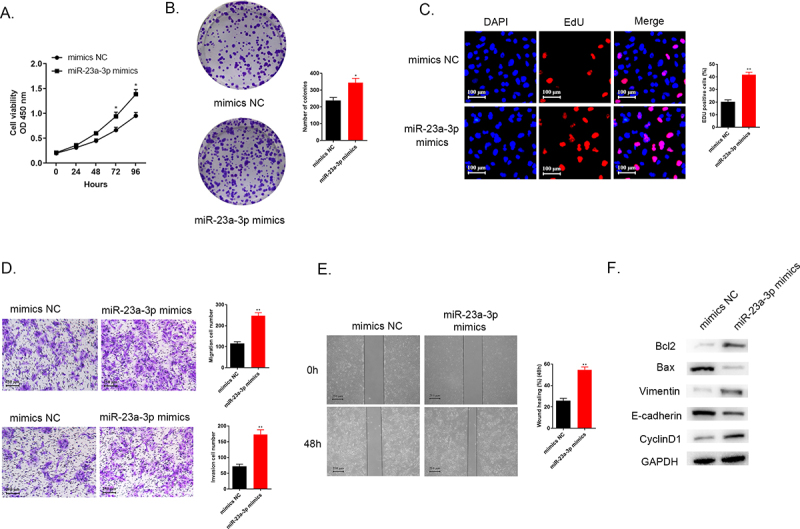
**Increasing miR-23a-3p expression was able to accelerate proliferation, invasion, as well as migration of cancer cells**. (a) We applied CCK-8 assay for detection of cell viability in RBE cells after transfection with miR-23a-3p mimics or mimics NC. (b-c) Colony formation as well as EdU assays examined proliferation ability of cells when miR-23a-3p overexpression, respectively. (d) Transwell assays aimed to observed cell invasion as well as migration when miR-23a-3p overexpression. (e) Wound healing assay detected cell migration when miR-23a-3p overexpression. (f) Quantitation of proteins correlated to apoptosis (Bax and Bcl-2), EMT pathway biomarkers correlated to tumor transition (Vinmentin and E-cadherin) and cell cycle protein (CyclinD1) were examined by Western blot.

Moreover, we explored the impacts of miR-23a-3p overexpression on RBE cells. Cell viability was detected with the help of CCK-8 assays, colony formation, as well as EdU assays. The data pointed out that higher miR-23a-3p level promoted cancer cell proliferation in comparison to the mimics NC group (Figure 3a–c). The findings of transwell assays and wound healing test demonstrated that the accelerated invasion as well as migration abilities were observed in RBE cells treated with miR-23a-3p mimics in comparison to mimcs NC group (Figure 3d–e). Figure 3f shows that up-regulated apoptosis regulators Bcl-2 level and down-regulated Bax level, increasing Vimentin expression and decreasing E-cadherin protein level and the elevating CyclinD1 level were observed in miR-23a-3p mimics group in comparison to mimics NC group. These findings illustrated that up-regulation of miR-23a-3p benefitted for cholangiocarcinoma cell proliferation, migration and invasion.

### MiR-23a-3p facilitated tumor development and metastasis in vivo

**Figure 4.**
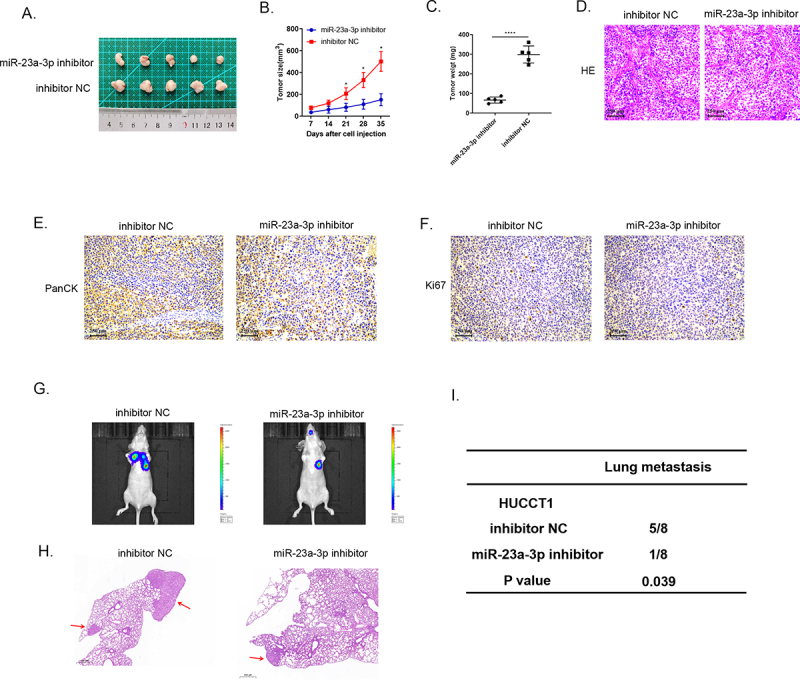
**Decreasing miR-23a-3p expression could inhibit growth, as well as metastasis of tumor in vivo**. (a) The image of tumor in miR-23a-3p inhibitor as well as inhibitor NC groups. (b) The tumor growth curve when miR-23a-3p been knockdown. (c) The average tumor weight when miR-23a-3p been knockdown. (d) HE staining of subcutaneous tumors. (e) Detected PanCK protein using IHC staining of subcutaneous tumors. (f) Down-regulation of Ki-67 in miR-23a-3p inhibitor group in comparison to inhibitor NC group. (g-i) Lung metastasis and HE staining results of tumor when miR-23a-3p been knockdown.

**Figure 5.**
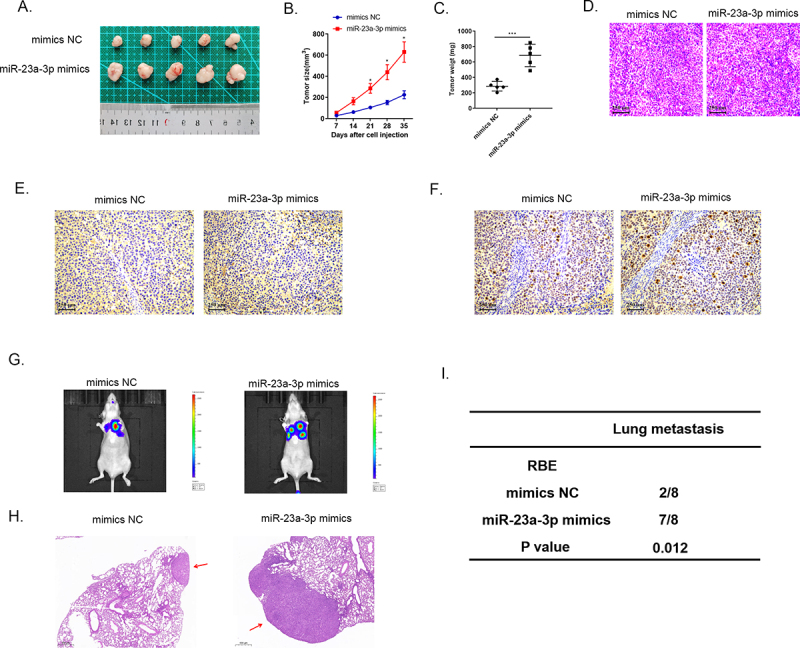
**MiR-23a-3p mimics promoted growth, as well as metastasis of tumor in vivo**. (a) The image of tumor in miR-23a-3p mimics as well as mimics NC groups. (b) The tumor growth curve when miR-23a-3p been upregulated. (c) The average tumor weight when miR-23a-3p was upregulated. (d) HE staining of subcutaneous tumors. (e) Detected PanCK protein using IHC staining of subcutaneous tumors. (f) Up-regulation of Ki-67 in miR-23a-3p mimics group in comparison to mimics NC group. (g-i) Lung metastasis and HE staining results of tumor when miR-23a-3p was upregulated.

Animal studies were utilized to specifically elucidate miR-23a-3p biological activity. We injected HUCCT1 cells transfected with miR-23a-3p inhibitor or inhibitor NC plasmids into nude mice subcutaneously, as well as RBE cells transfected with miR-23a-3p mimics or mimics NC plasmids. We recorded the volume of tumor every 7 days. At day 35, we handled all mice and collected the tumor tissues (Figures 4a and 5a). The tumor growth curves and the average tumor weights proved that silencing miR-23a-3p slowed down the growth of tumor (Figure 4b,c) and overexpressed miR-23a-3p boosted the growth of tumor in comparison to the control group (Figure 5b,c). Then, we resected the cholangiocarcinoma tissues and stained them with HE (Figures 4d and 5d) and detected PanCK protein by IHC staining (Figures 4e and 5e). Figures 4f and 5f demonstrate down-regulation of Ki-67 in the group of miR-23a-3p inhibitor in comparison to inhibitor NC group and up-regulation of Ki-67 in miR-23a-3p mimics group in comparison to mimics NC group. The six-week lung metastasis model was conducted through tail vein injection. The fluorescence image of mice and HE staining result exhibited that knockdown miR-23a-3p could prevent tumor metastasis (Figure 4g–i) while high level of miR-23a-3p accelerated the invasion as well as migration of tumor (Figure 5g–i). To sum up, these findings prompted that miR-23a-3p could serve as a cancer promoting gene, contributing to cholangiocarcinoma development in vivo.

### Exosome transmitted miR-23a-3p in cholangiocarcinoma cancer cells

**Figure 6.**
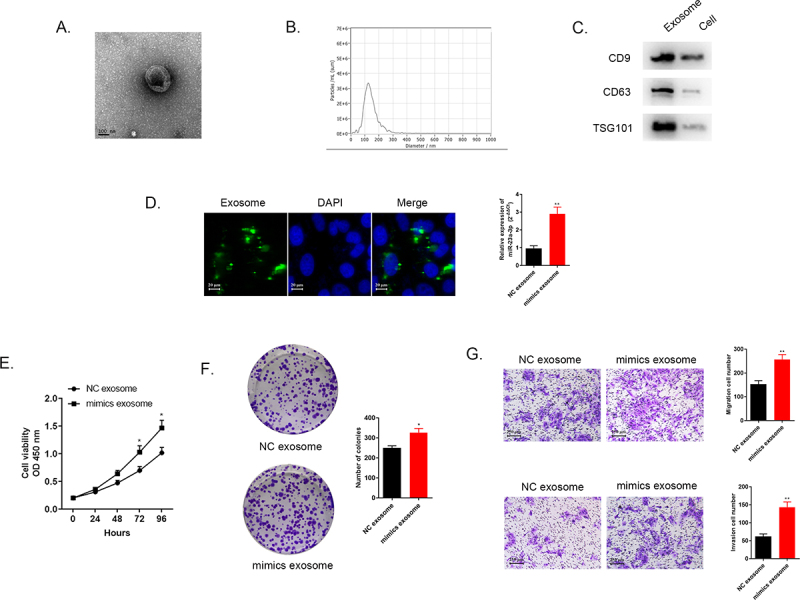
**Exosome transmitted miR-23a-3p and motivated proliferation, invasion, as well as migration of cholangiocarcinoma cancer cells**. (a) The morphology of exosomes was confirmed via electron microscopy. (b) The distribution of particle size was observed by NTA analysis. (c) Western blot detected exosome specific protein. (d) We labeled the exosomes extracted then cultured them with RBE cells. MiR-23a-3p level in RBE cells co-cultured with exosomes from HUCCT1 cells after transfection with miR-23a-3p mimics was greater than that in the mimics NC group. (e-f) After transfecting with mimics exosome or NC exosome in RBE cells, CCK-8 as well as colony formation assays aimed to confirm cell proliferation. (g) Transwell assays evaluated invasion as well as migration ability of transfected RBE cells.

Numerous researches have found that exosomes served as crucial mediators in cellular communication process [22,23]. Exosomal miRNAs were proved to be associated with cancer development and drug resistant [24,25]. Herein, we transfected HUCCT1 cells with miR-23a-3p mimics or mimics NC plasmid, and after that, we extracted and purified exosomes via differential centrifugation method. The morphological character of the separated exosome was observed under transmission electron microscope (Figure 6a). The distribution of the particle was about 100–200 nm identified by NTA analysis (Figure 6b). Western blot was applied for detection of specific protein markers of exosomes, CD9, CD63, as well as TSG101 (Figure 6c). Furthermore, we used membrane phospholipid dye PKH67 to mark the particles and then co-cultured with RBE cells. After 12 h, the image showed the successful merge. QRT-PCR examined miR-23a-3p expression level in co-incubation RBE cells. Figure 6d showed that miR-23a-3p turned out to be much higher in RBE cells with the uptake of exosomal miR-23a-3p mimics plasmid. These assays identified that miR-23a-3p could be uptook and transmitted by exosomes in cancer cells.

### Exosomal miR-23a-3p motivated cancer cells proliferation, migration and invasion

After the transfection with exosomal miR-23a-3p mimics or exosomal miR-23a-3p NC in RBE cells, related function assays need to be explored. The results of CCK-8 as well as colony formation assays indicated that exosomes with higher miR-23a-3p level promoted RBE cancer cell proliferation (Figure 6e–f). Transwell assays further certified that excessive exosomal miR-23a-3p facilitated cancer cell migration and invasion in comparison to miR-23a-3p NC group (Figure 6g). All these results stated that exosomal miR-23a-3p was able to motivate proliferation, invasion, as well as migration of cancer cells.

### MiR-23a-3p targeted to DNM3 with negative regulation and the inhibition of DNM3 expedited cancer cells proliferation, migration and invasion

**Figure 7.**
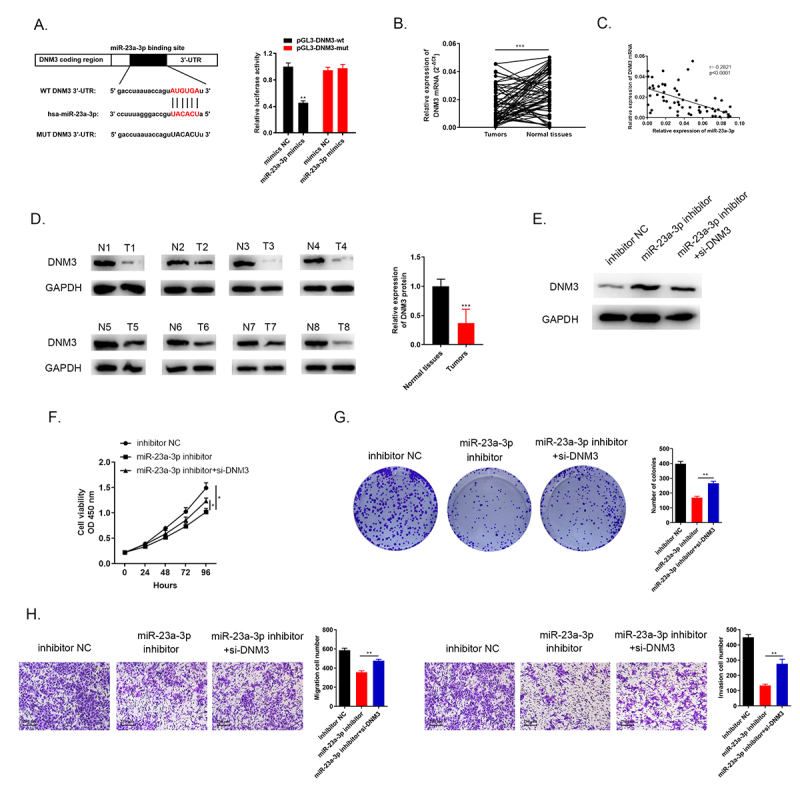
**miR-23a-3p targeted to DNM3 with negative regulation and the inhibition of DNM3 expedited cancer cells proliferation, migration and invasion**. (a) Bioinformatics analysis indicated there were complementary sequences between DNM3 and miR-23a-3p. The direct relationship between them was provided by the dual luciferase reporter assay. (b) DNM3 was downregulated confirmed via qRT-PCR in 60 pairs of cholangiocarcinoma tissues as well as normal tissues. (c) MiR-23a-3p was negatively correlated with DNM3 expression. (d) Western blot observed the suppressed DNM3 level in 8 pairs of cancer tissues. (e) The decreasing DNM3 level was found after transfection with miR-23a-3p inhibitor as well as si-DNM3. (f-g) CCK-8 as well as colony formation assays displayed the enhanced proliferation level of cells after silencing DNM3. (h) Tranwell assays exhibited that knockdown DNM3 could promote cell migration and invasion.

Through the bioinformatic database prediction, there were complementary sequences between DNM3 and miR-23a-3p (Figure 7a). The direct relationship between them was provided by the dual luciferase reporter assay. The luciferase activity of miR-23a-3p treated group was reduced compared to miR-NC group in pGL3-DNM3-wt cells while the luciferase activity in pGL3-DNM3-mut cells remained with no change between the two groups (Figure 7a). QRT-PCR showed DNM3 mRNA was downregulated in 60 pairs of cholangiocarcinoma tissues and normal tissues (Figure 7b). Pearson’s correlation analysis showed that miR-23a-3p was negatively correlated with DNM3 expression (Figure 7c). The DNM3 protein level in eight pairs of cholangiocarcinoma tissues was detected by Western blots, indicating that DNM3 was down-regulated (Figure 7d). In addition, we conducted three groups to evaluate the biological function of DNM3. We transfected HUCCT1 cells with inhibitor NC, miR-23a-3p inhibitor as well as miR-23a-3p inhibitor + si-DNM3, respectively. DNM3 level was lower in the group of miR-23a-3p inhibitor + si-DNM3 than in miR-23a-3p inhibitor group detected via Western blot (Figure 7e). Cell proliferation was enhanced by knockdown of DNM3 compared to miR-23a-3p inhibitor group via CCK-8 as well as colony formation assays (Figure 7f–g). The invasion as well as migration abilities of cell were also accelerated by silencing DNM3 measured by transwell assays (Figure 7h). As a result, these findings confirmed that miR-23a-3p targeted to DNM3 with negative regulation, the inhibition of DNM3 expedited cancer cell proliferation, migration and invasion.

## Discussion

Cholangiocarcinoma is one of the most common liver and biliary system tumor after hepatocellular carcinoma [26]. The current mechanism is unclear and the efficient cure methods are urgent. MiRNAs are closely involved in many cancer development and have been identified as promising biomarkers for the targets of cancer diagnosis as well as treatment. For instance, elevated expression level of miR-99b was correlated to the poorer overall survival time in cholangiocarcinoma patients [27,28]. Upregulation of miR-1182 could induce cancer cell apoptosis, repressed cholangiocarcinoma proliferation as well as metastasis both in vitro and in vivo [29]. So far, various miRNAs exert different biological functions in cholangiocarcinoma, and the specific regulation mechanism needs to be scrutinized to provide novel direction. Initially, exosomes were regarded as redundant membrane proteins that were released in the process of cell maturation via regulating membrane function, and organelles that remove cell debris and eliminate cell surface molecules [30,31]. A growing number of researches have indicated that exosomes are closely involved in mediating inflammatory response, cell proliferation, regulating extracellular microenvironment and inducing immune response. Nowadays, exosomes are recognized as biomarkers and prognostic factors for disease, and have potential clinical importance as drug or gene delivery vectors [32].

## Conclusion

In conclusion, we firstly observed that the over-expression of miR-23a-3p in CCA tissues as well as cell lines via bioinformatics prediction. Inhibition of miR-23a-3p could prevent the progression of CCA both in vivo and in vitro. Furthermore, it was identified CCA cells transferred miR-23a-3p through exosomes, and that exosomal miR-23a-3p could promote cancer cell growth and metastasis. Furthermore, DNM3 was found to be a promising target of miR-23a-3p in CCA. The knockdown of DNM3 accelerated cancer cell development. Collectively, our findings firstly pointed out that exosomal miR-23a-3p was conducive to the progression of CCA by interaction with DNM3, miR-23a-3p might be a new biomarker for cholangiocarcinoma treatment.

## Supplementary Material

Supplemental Material
