# Supplementary material for: Exosomal microRNA-23a-3p contributes to the progression of cholangiocarcinoma by interaction with Dynamin3
Source: Bioengineered. 2022 Feb 24;13(3):6208–21. doi: 10.1080/21655979.2022.2037249 (PMC8973721; doi:10.1080/21655979.2022.2037249)

Bcl2 for Fig.3H and 2H





Bax for Fig.3H and 2H





Vimentin for Fig.3H and 2H





E-cadherin for Fig.3H and 2H





CyciinD1 for Fig.3H and 2H





GAPDH for Fig.3H and 2H


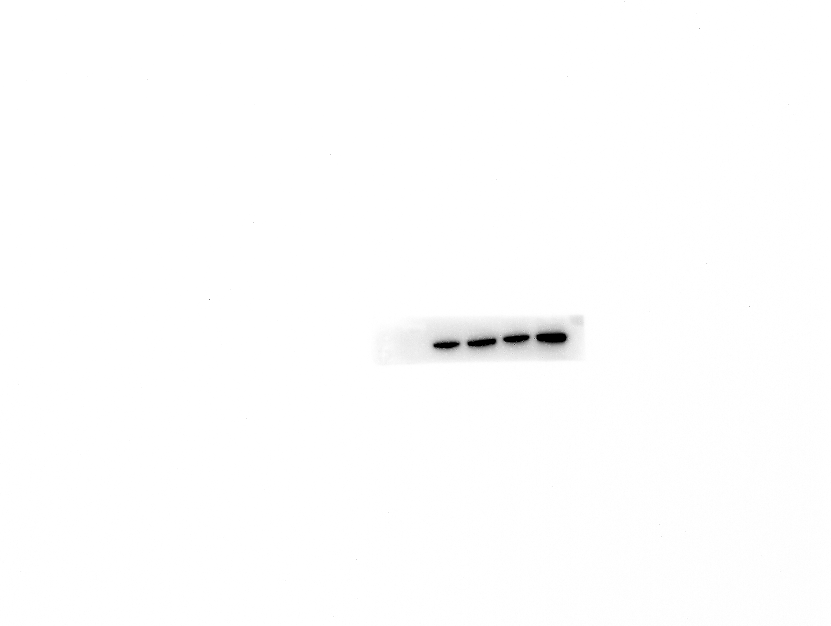


CD9 for Fig.6C


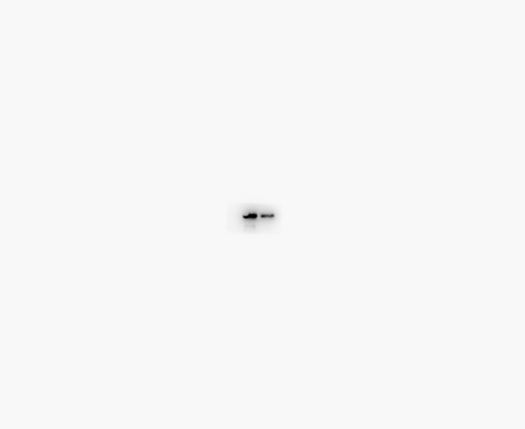


CD63 for Fig.6C


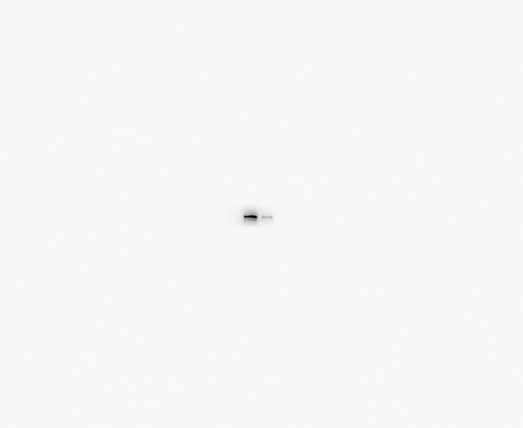


TSG101 for Fig.6C


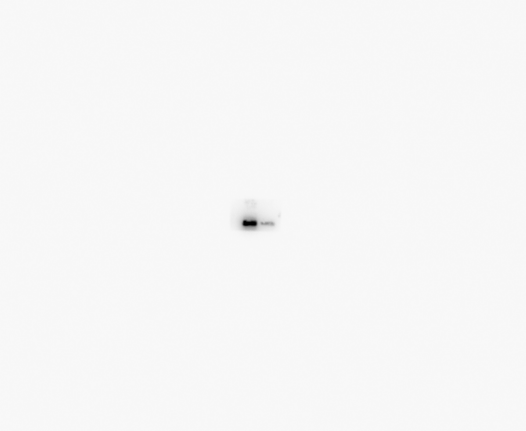


DNM3 for Fig.7D


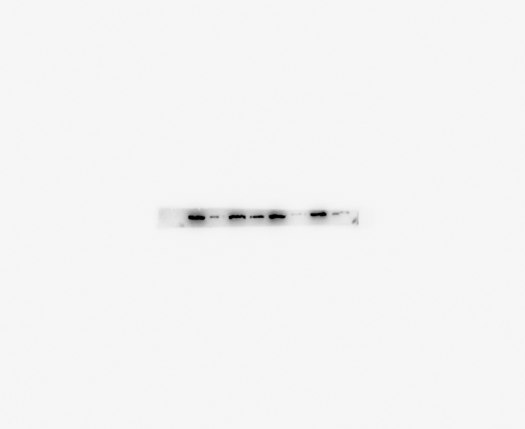


DNM3 for Fig.7D


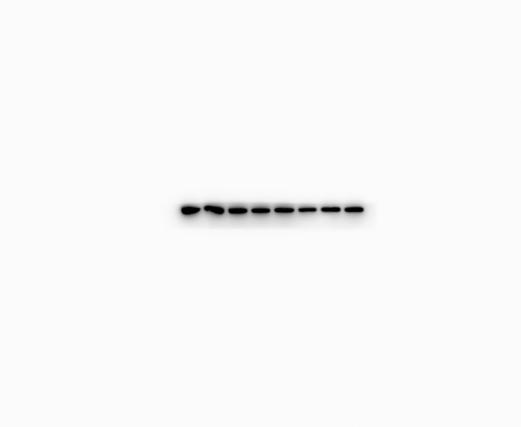


GAPDH For Fig.7D


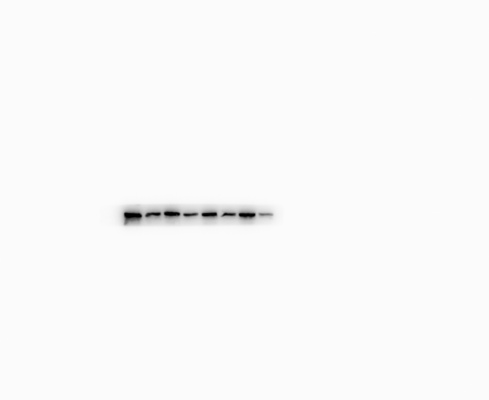


GAPDH For Fig.7D


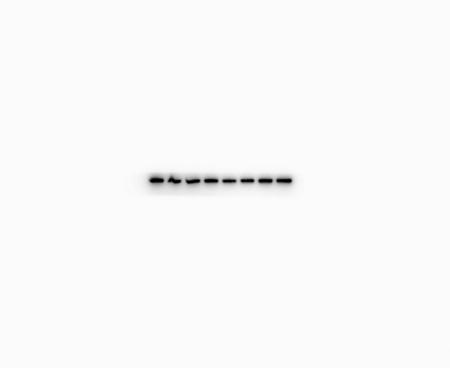


DNM3 for Fig.7E


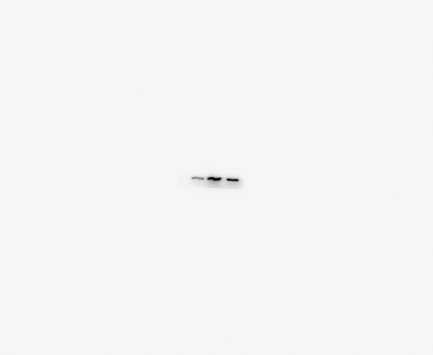


GAPDH for Fig.7E


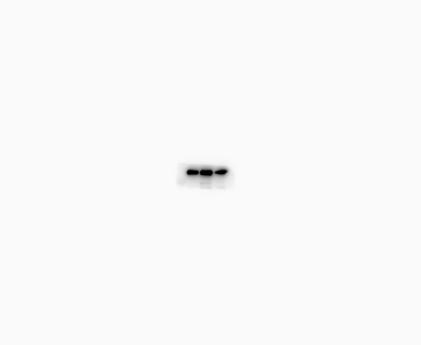

Supplement: Supplemental Material [file KBIE_A_2037249_SM8617.docx]
